# Effect of a Multicomponent Intervention Delivered on a Web-Based Platform on Hypertension Control: A Cluster Randomized Clinical Trial

**DOI:** 10.1001/jamanetworkopen.2022.45439

**Published:** 2022-12-07

**Authors:** Haoqi Zhou, Xin Wang, Ying Yang, Zuo Chen, Linfeng Zhang, Congyi Zheng, Lan Shao, Ye Tian, Xue Cao, Zhen Hu, Yixin Tian, Lu Chen, Jiayin Cai, Runqing Gu, Zengwu Wang

**Affiliations:** 1 Division of Prevention and Community Health, National Center for Cardiovascular Disease, National Clinical Research Center of Cardiovascular Disease, State Key Laboratory of Cardiovascular Disease, Fuwai Hospital, Peking Union Medical College & Chinese Academy of Medical Sciences, Beijing, China; 2 Department of Biostatistics, Peking University, Beijing, China; 3 Cardiovascular Center, Beijing Huaxin Hospital, the First Hospital of Tsinghua University, Beijing, China; 4 School of Population Medicine and Public Health, Peking Union Medical College & Chinese Academy of Medical Sciences, Beijing, China

## Abstract

**Importance:**

The prevalence of hypertension is high and still increasing across the world, while the control rate remains low in many countries. Emerging technology, such as telemedicine, may offer additional support to change the unsatisfactory situation.

**Objective:**

To establish a multicomponent intervention delivered on a web-based telemedicine platform and oriented with the Chinese hypertension management guidelines and to evaluate the effect of the intervention on blood pressure (BP) control for patients with hypertension.

**Design, Setting, and Participants:**

This cluster randomized clinical trial of a hypertension management program was conducted at 66 community health centers in China from October 1, 2018, to May 31, 2020, with a 12-month follow-up. Patients with hypertension were blinded to randomization and were randomized to either the intervention group or control group. Hypertension was diagnosed at mean systolic BP (SBP) and diastolic BP (DBP) readings higher than 140 and 90 mm Hg or with use of antihypertensive medication. Evaluation of the intervention effect was based on the principle of modified intention to treat.

**Interventions:**

Multicomponent intervention was delivered on a web-based platform and consisted of a primary prevention program for cardiovascular disease and standardized management for hypertension.

**Main Outcomes and Measures:**

The primary outcome was the change in BP control rate (SBP and DBP levels <140 and 90 mm Hg, or <130 and 80 mm Hg for patients with diabetes) from baseline to the 12-month follow-up among patients with hypertension in the intervention and control groups.

**Results:**

A total of 4118 patients (mean [SD] age, 61.6 [9.4] years; 2265 women [55.0%]) were included in the analysis, with 2985 in the intervention group and 1133 in the control group. The BP control rate at baseline was 22.8% in the intervention group and 22.5% in the control group. After 12 months of the intervention, the BP control rate for the intervention group compared with the control group was significantly higher (47.4% vs 30.2%; odds ratio, 1.18; 95% CI, 1.13-1.24; *P* < .001). The intervention effect on SBP level was –10.1 mm Hg (95% CI, –11.7 to –8.5 mm Hg; *P* < .001) and on DBP level was –1.8 mm Hg (95% CI, –2.8 to –0.8 mm Hg; *P* < .001).

**Conclusions and Relevance:**

Results of this trial showed that a multicomponent intervention delivered on a web-based platform improved BP control rate and lowered BP level more than usual care alone. Such a telemedicine program may provide a new, effective way to treat patients with hypertension in the community and may generate public health benefits across diverse populations.

**Trial Registration:**

Chinese Clinical Trial Registry Identifier: ChiCTR1800017791

## Key Points

**Question:**

Can a multicomponent intervention delivered on a web-based platform and oriented with the Chinese hypertension management guidelines improve blood pressure control among patients with hypertension in the community?

**Findings:**

In this cluster randomized clinical trial of 4118 patients with hypertension, patients in the intervention group achieved better blood pressure control than the control group.

**Meaning:**

Findings of this study indicate that a multicomponent intervention delivered on a web-based platform might be an effective way to manage patients with hypertension in the community.

## Teaser

This cluster randomized clinical trial examines the effect of a web-based intervention on hypertension management in China.

## Introduction

Cardiovascular disease (CVD) is the leading cause of death globally, and about one-third of deaths every year are caused by CVD.^1,2^ Hypertension is the most common risk factor for CVD, and evidence from previous studies has confirmed the association of reduced risk of cardiovascular events and death with lower blood pressure (BP).^3,4^ The risk of stroke and myocardial infarction increased by 53% and 31%, respectively, due to the elevated BP in Asia Pacific study populations.^5^ According to the most recent national hypertension survey in China,^6^ approximately 244.5 million Chinese adults 18 years or older had hypertension, but among individuals with hypertension, only 15.3% had adequate BP control. Such a huge number of patients with hypertension has brought a great burden to the medical and health care systems in China.^7^

Based on previous work, traditional interventions concerning health education, control of risk factors, and lifestyle modifications have succeeded in lowering the BP level and improving the control rate of patients with hypertension, whether in the community^8^ or workplaces.^9^ However, poor adherence to treatment and low control rate still existed among patients with hypertension,^10^ which required further improvement. It is a big challenge to implement such interventions nationwide because of the high costs of organization and the limited medical resources available in China.^11^ Given the high burden of hypertension and the aging population in China, novel and effective interventions are urgently needed to improve BP control.

Emerging technology, such as telemedicine, can potentially help people in self-management by delivering health education and facilitating interaction between patients and primary care physicians. In addition, personalized recommendations and interventions would be generated according to basic characteristics and disease conditions. Studies have shown that telemedicine, including BP telemonitoring and text message intervention, is associated with reduced systolic BP (SBP) and diastolic BP (DBP) levels.^12,13^ However, heterogeneity exists among previous studies,^14,15^ and the interventions are relatively limited and multicomponent interventions are lacking. Studies have found that hypertension is associated with a range of risk factors, such as excessive salt consumption,^16^ less physical activity,^17^ and obesity,^18^ which determined that co-interventions are essential to improved BP control. Telemedicine also may offer even more possibilities, bring diverse interventions together, and simplify the implementation. Therefore, in this trial, we aimed to establish a multicomponent intervention delivered on a web-based telemedicine platform and oriented with the Chinese hypertension management guidelines^19^ and to evaluate the effect of the intervention on BP control for patients with hypertension.

## Methods

### Study Design

In this cluster randomized clinical trial, the community health center was the unit of randomization. The trial was approved by the institutional review boards of Fuwai Hospital and each of the 22 subcenters. All participants provided written informed consent before screening. We followed the Consolidated Standards of Reporting Trials (CONSORT) reporting guideline. The study protocol is provided in Supplement 1, and the CONSORT flow diagram is provided in eFigure 1 in Supplement 2.

**Figure 1.**
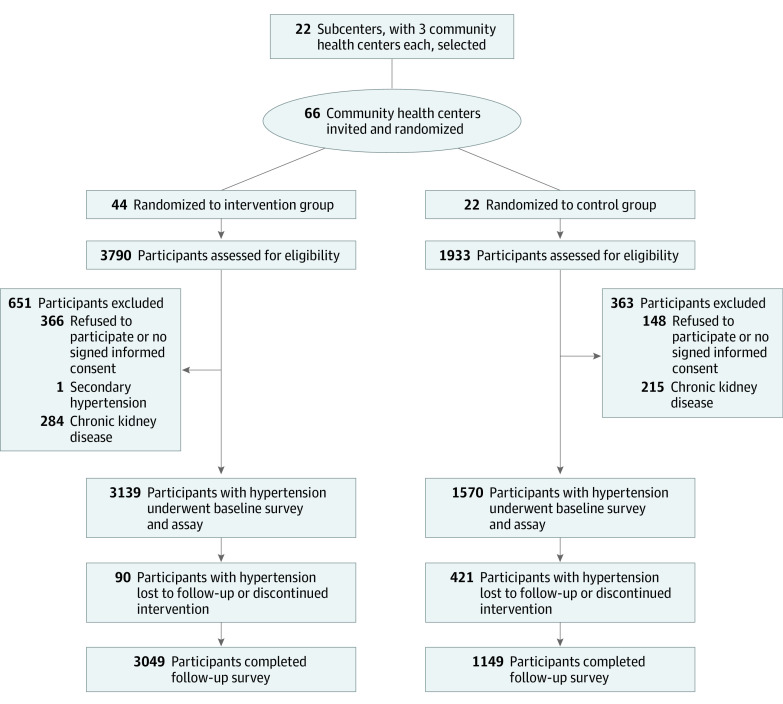
Screening, Randomization, and Follow-up Flowchart

Twenty-two medical institutions, including general hospitals and centers for disease control and prevention (12 in towns and 10 in counties), were selected as subcenters (Figure 1). Three community health centers with comparable medical level, population size, and economic development level were invited from each subcenter. Patients from 2 of these centers were randomized to the intervention group and patients from 1 of these centers were randomized to the control group after completion of a cross-sectional survey. The randomization was undertaken by a statistician (Z.C.) using SAS, version 9.4 (SAS Institute Inc) to generate a random sequence in the coordinating center, which was not involved in the trial and was blind to the communities.

### Participants

After randomization, from October 1, 2018, to May 31, 2019, participants were recruited and screened for eligibility. The completed inclusion and exclusion criteria are included in the protocol (Supplement 1). The present study focused on hypertension, and thus the main inclusion criteria for individuals were as follows: hypertension diagnosis, with mean SBP and DBP levels higher than 140 and 90 mm Hg or use of antihypertensive medication; local area residency; and signed consent form and agreement to receive the 12-month intervention. Individuals with secondary hypertension, acute myocardial infarction or stroke within 3 months, chronic kidney disease, pregnancy, serious disease (eg, cancer), and life expectancy of less than 1 year were excluded. Race and ethnicity data were not collected because most participants were of Han nationality.

### Intervention

Only patients who were randomized to the intervention group received the intervention. A 12-month multicomponent intervention program aimed at improving hypertension control was developed on the basis of recommendations from the 2017 Chinese hypertension management guidelines^19^ and a previous trial.^9^ The complete intervention is available in the study protocol in Supplement 1. The intervention incorporated the primary prevention program for CVD and the standardized management for hypertension.

The primary prevention program was aimed at improving health awareness and empowering patients with hypertension to manage CVD risk factors and included health education and lifestyle modification. Health education was delivered via leaflets about general disease knowledge (risk factors and prevention) of hypertension and BP control. Lifestyle modification included education on nutrition and choosing a reasonable diet (low-salt, low-fat, and low-calorie), recommendations to increase physical activity, and strategies for weight loss and cigarettes or alcohol cessation. Patients in the intervention group were also encouraged to self-monitor at home.

The standardized management for hypertension consisted of classification of BP, stratification of CVD risk, setting targets for BP control, drug treatment, and follow-up management plan. For the treatment, the principles of low-dose initiation, long-acting medication, combination therapy, and individualization were used according to the Chinese hypertension management guidelines.^19^ Patients in the intervention group were followed up monthly in the designated community health center according to the follow-up plan for adherence reinforcement of healthy lifestyle and treatment. The treatment could be modified as necessary by trained primary care physicians according to the study protocol (Supplement 1).

To facilitate the implementation of the intervention, we established a web-based platform oriented with the Chinese hypertension management guidelines^19^ to aid primary care physicians; a detailed description of the platform is found in the protocol in Supplement 1 and eFigure 2 in Supplement 2. Briefly, the primary care physicians could log into the platform and access all of the study questionaries. At baseline, a digital record of social characteristics, lifestyle, history of disease, and physical examination based on cross-sectional survey data was created for each patient in the intervention group and stored on the platform. Then, automated BP classification of the baseline profile and the schedule for follow-up were generated according to a prespecified algorithm. As the follow-up date neared, the platform alerted the primary care physician to start a routine visit, and a telephone call to the patient was made. During the visit, compliance with medicine therapy, lifestyle changes, BP level, and adverse events was evaluated and entered into the platform. Based on the result of automated evaluation, the primary care physician provided personalized health education and advice on medication to the patient either face to face or through text messages.

At the beginning of this trial, all of the community primary care physicians who participated received standard training on how to use the platform. All of the patients in the intervention group were asked to provide their own (or a family member’s, if the participant had no telephone) telephone number to receive the text messages or calls from the primary care physicians. Patients were informed that they could withdraw from the study at any time they wanted. Researchers from the coordinating center (including H.Z., X.W., Y.Y., and Z.W.) visited each subcenter regularly for supervision to ensure the appropriate implementation of the intervention.

Patients who were randomized to the control group received only usual care, which consisted of existing Essential Public Health Service,^20^ with routine care for prevention or treatment of hypertension by community primary care physicians. Patients in the control group were seen only at baseline and at end of the trial, and they received neither the intervention nor the scheduled follow-up for the trial.

### Outcomes

At the time of participant recruitment, baseline data of the intervention and control groups, including sociodemographic characteristics, lifestyle, history of disease, and medical treatment, were collected by trained staff at face-to-face visits using a standardized questionnaire. At each follow-up visit, BP levels and information on lifestyle factors and medication use were also collected from the intervention group.

Blood pressure was measured on the right arm at heart level in a seated position using an automatic digital BP monitoring device (Omron HBP-1300; Omron). All BP measurements were conducted by trained staff, and at least 5 minutes of rest before measurement and 1-minute interval between readings were required. If the difference between the 2 measurements was greater than 5 mm Hg for SBP or DBP, a third measurement was conducted. The last 2 readings were recorded and used for the analyses. The definition of BP control was mean SBP and DBP readings lower than 140 and 90 mm Hg (<130 and 80 mm Hg for patients with diabetes). Stage 1 of hypertension was defined as SBP level of 140 to 159 mm Hg and DBP level of 90 to 99 mm Hg, stage 2 as SBP level of 160 to 179 mm Hg and DBP level of 100 to 109 mm Hg, and stage 3 as SBP level of 180 mm Hg or higher and DBP level of 110 mm Hg or higher.^19^

Body mass index (BMI) was calculated as weight in kilograms divided by height in meters squared. A BMI between 24 and 27.9 was defined as overweight, and a BMI of 28 or higher was defined as obesity.^21^ According to a previous study,^22^ individuals who smoked at least 1 cigarette per day were regarded as current smokers, and those who consumed alcohol at least once a week were current drinkers; regular exercise was defined as physical exercise performed for more than 30 minutes at a time at least 3 times a week.

Outcomes were measured as changes from baseline to 12 months. The primary outcome was the change in percentage of patients with controlled BP (SBP and DBP readings of <140 and 90 mm Hg, or <130 and 80 mm Hg for patients with diabetes). The secondary outcomes were mean changes in SBP and DBP readings, the proportion of overweight or obesity, drug use, current smoking, current drinking, and regular exercise. Values (at 12 months minus at baseline) of the intervention group minus values of the control group were calculated to present the intervention effect for continuous outcomes. Odds ratios (OR) and 95% CIs of the intervention group compared with the control group were reported for dichotomous outcomes. Adverse events, including acute myocardial infraction, coronary artery bypass graft, stroke, and all-cause death, were reported.

### Statistical Analysis

Given the intervention benefits, we used a 2:1 ratio to recruit patients. A sample size of 4500 (3000 in the intervention group, and 1500 in the control group) was estimated with a 2.5% type I error, intracluster correlation coefficient of 0.01,^23^ and 80% retention to provide a 90% power to detect a 20% absolute difference in BP control rate at the 12-month follow-up between the intervention group and control group. It was recommended that 75 patients be recruited at baseline from each community health center.

Evaluation of the intervention effect was based on the principle of modified intention to treat. Patients who completed their last follow-up visit at 12 months and had complete data for the primary outcome and other variables were included in the analysis. As prespecified in the statistical plan, for the primary outcome analyses, the intervention effect on BP control rate over time, and changes of BP readings from baseline, we used mixed-effects regression analyses models that included a random cluster effect (community health center). The models were adjusted for baseline variables, including age, sex, region, BMI group, smoking status, drinking status, regular exercise, and CVD family history. The heterogeneity of community health centers intervention was also evaluated. To handle the missing data on outcome and other covariates, a multiple imputation method was used for sensitivity analyses for robust primary outcome analyses (Supplement 1).

An exploratory analysis was also conducted in predefined subgroups, including sex, age, educational level, region, BMI group, and CVD family history. SAS, version 9.4 and R, version 4.0 (R Foundation for Statistical Computing) were used to perform the analyses. A 2-sided *P* < .05 was considered to be statistically significant.

## Results

We analyzed 4118 patients with complete data (2985 in the intervention group and 1133 in the control group), who had a mean (SD) age of 61.6 (9.4) years; 2265 patients were women (55.0%) and 1853 were men (45.0%). The mean SBP and DBP levels were 148.6 and 85.2 mm Hg, and 681 patients (22.8%) in the intervention group and 255 patients (22.5%) in the control group had controlled BP. Characteristics of the patients in the intervention and control groups were well balanced except for region (Table 1).

**Table 1.** Baseline Characteristics of Participants

	Participants, No. (%)		
	Total (n = 4118)	Control group (n = 1133)	Intervention group (n = 2985)
Age, mean (SD), y	61.6 (9.4)	61.6 (9.4)	61.6 (9.4)
Sex			
Female	2265 (55.0)	610 (53.8)	1655 (55.4)
Male	1853 (45.0)	523 (46.2)	1330 (44.6)
Smoking status			
Female	24 (1.1)	4 (0.7)	20 (1.2)
Male	609 (32.9)	159 (30.4)	450 (33.8)
Drinking status			
Female	87 (3.84)	29 (4.8)	58 (3.5)
Male	650 (35.1)	166 (31.7)	484 (36.4)
BMI group			
Healthy	1158 (28.5)	300 (26.7)	858 (29.2)
Overweight	1789 (44.0)	509 (45.2)	1280 (43.5)
Obesity	1120 (27.5)	316 (28.1)	804 (27.3)
SBP level, mean (SD), mm Hg	148.6 (18.3)	147.5 (17.9)	148.9 (18.5)
DBP level, mean (SD), mm Hg	85.2 (11.4)	85.5 (11.3)	85.1 (11.5)
BP control rate	936 (22.7)	255 (22.5)	681 (22.8)
CVD family history			
No	2557 (62.1)	728 (64.3)	1829 (61.3)
Yes	1561 (37.9)	405 (35.7)	1156 (38.7)
No. of antihypertensive medications^a^			
0	1481 (36.0)	422 (37.3)	1059 (35.5)
1	1817 (44.1)	479 (42.3)	1338 (44.8)
2	663 (16.1)	191 (16.9)	472 (15.8)
>2	157 (3.8)	41 (3.6)	116 (3.9)
Region			
West	1162 (28.2)	324 (28.6)	838 (28.1)
Central	999 (24.3)	327 (28.9)	672 (22.5)
East	1957 (47.5)	482 (42.5)	1475 (49.4)

Abbreviations: BMI, body mass index; BP, blood pressure; CVD, cardiovascular disease; DBP, diastolic blood pressure; SBP, systolic blood pressure.

^a^ Numbers of medications were defined as follows: 0 for no drug use; 1 for 1 monotherapy; 2 for 2-drugs free combination or single-pill combination consisting of 2 active ingredients; >2 for more than 2-drugs free combination or single-pill combination consisting of more than 2 active ingredients.

Between October 1, 2018, and May 31, 2020, a total of 4709 patients with hypertension from 22 subcenters were invited at baseline (Figure 1). The basic characteristics for all patients enrolled at baseline were shown in eTable 1 in Supplement 2. A total of 4198 patients completed the 12-month follow-up, and 511 patients (10.9%) were lost to follow-up. Comparison of patients at follow-up and those lost to follow-up is provided in eTable 2 in Supplement 2, and the difference between patients who were included and those lost to follow-up was not significant in terms of age, sex, BMI, BP levels, BP control rate, and proportion receiving antihypertensive medication.

**Figure 2.**
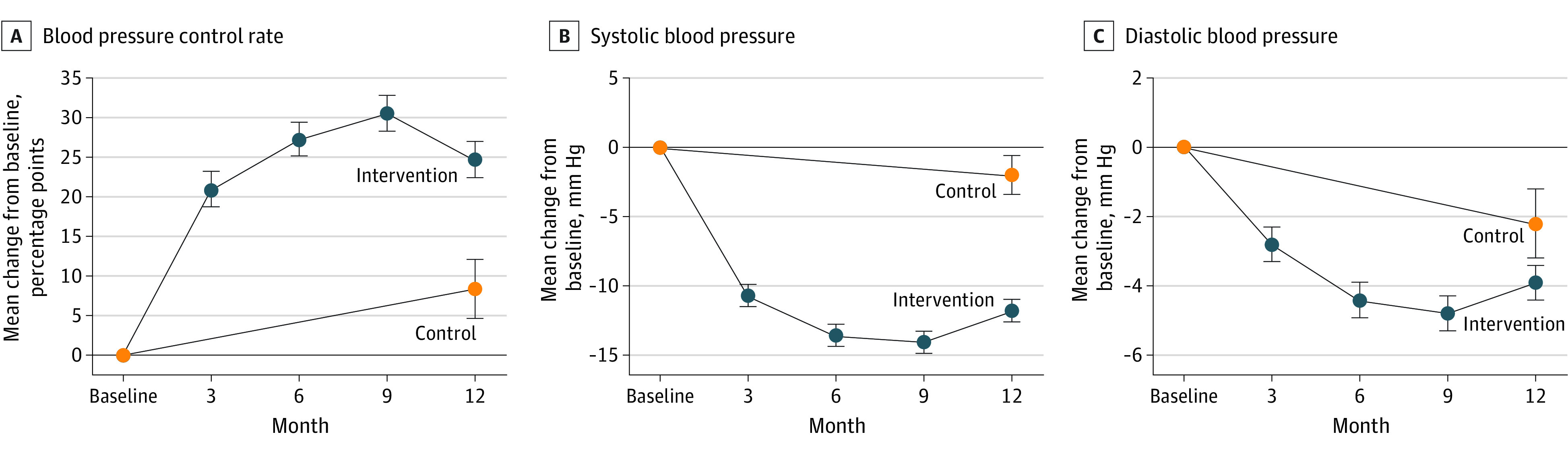
Mean Change in Blood Pressure Control Rate and Systolic and Diastolic Blood Pressure The horizontal lines at 0 indicate no change in control rate or blood pressure from baseline to end of follow-up; error bars indicate the 95% CIs.

From baseline to the first 3 months of intervention, a sharp increase of the BP control rate was observed in the intervention group (from 22.8% to 44.5%) and then a gradual increase throughout the study period (to 47.4%) (Figure 2A). The SBP level was significantly reduced from 148.9 mm Hg at baseline to 136.9 mm Hg at 12 months in the intervention group (*P* < .001) and from 147.5 mm Hg at baseline to 145.6 mm Hg at 12 months in the control group (*P* < .001) (Figure 2B). A similar pattern was found in DBP level, decreasing from 85.1 mm Hg to 81.1 mm Hg in the intervention group and from 85.5 mm Hg to 83.3 mm Hg in the control group (*P* < .001) (Figure 2C and eTable 3 in Supplement 2).

The BP control rate reached 47.4% in the intervention group and 30.2% in the control group, and the intervention effect was significant (OR, 1.18; 95% CI, 1.13-1.24; *P* < .001). The intervention’s effect on hypertension control was consistent across sex, age, educational level, region, BMI group, CVD family history, and antihypertensive medication use in the predefined subgroup analysis except for patients with a college degree or higher (Table 2). Significant random cluster effect (intraclass correlation coefficient, 0.07; *P* < .001) was observed in the study, illustrating the hierarchical nature of BP level and corresponding data from different subcenters. Patients with uncontrolled BP at baseline were more likely to have controlled BP at the 12-month visit in the intervention group vs the control group (OR, 1.17; 95% CI, 1.13-1.21; *P* < .001) (eTable 4 in Supplement 2).

**Table 2.** Blood Pressure Control Rate for Participants

Characteristic	Control group (n = 1133)				Intervention group (n = 2985)				Intervention effect^a^	
	No., %			OR (95% CI)	No., %			OR (95% CI)	OR (95% CI)	*P* value
	Baseline	12-Mo follow-up	Change from baseline (95% CI)		Baseline	12-Mo follow-up	Change from baseline (95% CI)			
Overall	255 (22.5)	342 (30.2)	7.7 (3.9 to 11.4)	1.08 (1.04 to 1.12)	681 (22.8)	1415 (47.4)	24.6 (22.3 to 26.9)	1.28 (1.25 to 1.31)	1.18 (1.13 to 1.24)	<.001
Sex										
Male	118 (22.6)	160 (30.6)	8.0 (2.5 to 13.5)	1.08 (1.03 to 1.15)	300 (22.6)	625 (47.0)	24.4 (21.0 to 27.9)	1.28 (1.23 to 1.32)	1.18 (1.10 to 1.26)	<.001
Female	137 (22.5)	182 (29.8)	7.4 (2.3 to 12.5)	1.08 (1.02 to 1.13)	381 (23.0)	790 (47.7)	24.7 (21.6 to 27.8)	1.28 (1.24 to 1.32)	1.19 (1.12 to 1.26)	<.001
Age, y										
<65	164 (24.3)	201 (29.8)	5.5 (0.6 to 10.4)	1.06 (1.01 to 1.11)	406 (23.4)	872 (50.3)	26.9 (23.8 to 29.9)	1.31 (1.27 to 1.35)	1.24 (1.17 to 1.31)	<.001
≥65	91 (19.8)	141 (30.7)	10.9 (5.1 to 16.7)	1.12 (1.05 to 1.18)	275 (22.0)	543 (43.4)	21.3 (17.8 to 24.8)	1.24 (1.19 to 1.28)	1.11 (1.04 to 1.19)	<.001
Educational level										
≤Middle school	177 (20.6)	240 (27.9)	7.3 (3.1 to 11.6)	1.08 (1.03 to 1.12)	521 (22.7)	1032 (45.0)	22.3 (19.7 to 24.9)	1.25 (1.22 to 1.28)	1.16 (1.10 to 1.22)	<.001
≤High school diploma	69 (27.7)	92 (36.9)	9.2 (1.1 to 17.4)	1.10 (1.01 to 1.19)	146 (23.2)	350 (55.6)	32.4 (27.3 to 37.6)	1.38 (1.31 to 1.46)	1.26 (1.15 to 1.39)	<.001
≥College degree	9 (39.1)	10 (43.5)	4.3 (−23.2 to 31.9)	1.04 (0.79 to 1.38)	14 (21.9)	33 (51.6)	29.7 (13.2 to 46.2)	1.35 (1.14 to 1.59)	1.29 (0.93 to 1.78)	.12
Region										
West	89 (27.5)	121 (37.3)	9.9 (2.7 to 17.0)	1.10 (1.03 to 1.19)	204 (24.3)	455 (54.3)	30.0 (25.5 to 34.4)	1.35 (1.29 to 1.41)	1.22 (1.12 to 1.33)	<.001
Central	65 (19.9)	92 (28.1)	8.3 (1.2 to 15.3)	1.09 (1.01 to 1.16)	195 (29.0)	410 (61.0)	32.0 (27.1 to 36.9)	1.38 (1.31 to 1.45)	1.27 (1.16 to 1.38)	<.001
East	101 (21.0)	129 (26.8)	5.8 (0.3 to 11.3)	1.06 (1.00 to 1.12)	282 (19.1)	550 (37.3)	18.2 (15.0 to 21.3)	1.20 (1.16 to 1.24)	1.13 (1.06 to 1.21)	<.001
BMI group										
Healthy	87 (29.0)	131 (38.9)	9.9 (2.5 to 17.2)	1.10 (1.03 to 1.19)	233 (27.2)	508 (53.3)	26.1 (21.8 to 30.5)	1.30 (1.24 to 1.36)	1.18 (1.08 to 1.28)	<.001
Overweight	108 (21.2)	154 (30.7)	9.5 (3.8 to 15.1)	1.10 (1.04 to 1.16)	311 (24.3)	593 (47.9)	23.6 (20.0 to 27.1)	1.27 (1.22 to 1.31)	1.15 (1.08 to 1.23)	<.001
Obesity	58 (18.4)	54 (19.1)	0.8 (−5.9 to 7.5)	1.01 (0.94 to 1.08)	129 (16.0)	285 (39.8)	23.8 (19.5 to 28.0)	1.27 (1.22 to 1.32)	1.26 (1.16 to 1.36)	<.001
CVD family history										
No	155 (21.3)	253 (29.4)	8.1 (3.5 to 12.7)	1.08 (1.04 to 1.13)	458 (25.0)	1120 (47.9)	22.9 (20.0 to 25.7)	1.26 (1.22 to 1.29)	1.16 (1.10 to 1.22)	<.001
Yes	100 (24.7)	89 (32.7)	8.0 (1.3 to 14.8)	1.08 (1.01 to 1.16)	223 (19.3)	295 (45.6)	26.3 (22.1 to 30.5)	1.30 (1.25 to 1.36)	1.20 (1.11 to 1.30)	<.001
Antihypertensive medication use										
No	79 (23.5)	80 (23.8)	0.3 (−6.3 to 6.9)	1.00 (0.94 to 1.07)	133 (18.3)	286 (39.3)	21.0 (16.6 to 25.5)	1.23 (1.18 to 1.29)	1.23 (1.14 to 1.33)	<.001
Yes	176 (22.1)	262 (32.9)	10.8 (6.3 to 15.3)	1.11 (1.06 to 1.17)	548 (24.3)	1129 (50.0)	25.7 (23.0 to 28.4)	1.29 (1.26 to 1.33)	1.16 (1.10 to 1.22)	<.001

Abbreviations: BMI, body mass index; CVD, cardiovascular disease.

^a^ Baseline variables were used for adjustment.

From baseline to the 12-month follow-up, the mean SBP level decreased 12.0 mm Hg (95% CI, –12.8 to –11.1 mm Hg; *P* < .001) in the intervention group vs 1.9 mm Hg (95% CI, –3.3 to –0.5 mm Hg; *P* = .006) in the control group (Table 3); the mean reduction in SBP level was 10.1 mm Hg greater in the intervention group than in the control group (95% CI, –11.7 to –8.5 mm Hg; *P* < .001). For DBP level, the mean reduction of –1.8 mm Hg (95% CI, –2.8 to –0.8 mm Hg; *P* < .001) was greater in the intervention group. Net reductions in SBP and DBP levels across sex, age, educational level, region, BMI group, and CVD family history in predefined subgroup analysis are provided in eFigure 3 in Supplement 2. The ORs for the intervention effect were 0.90 (95% CI, 0.86-0.94; *P* < .001) for stage 1 hypertension, 0.89 (95% CI, 0.86-0.93; *P* < .001) for stage 2 hypertension, and 0.94 (95% CI, 0.93-0.95; *P* < .001) for stage 3 hypertension (Table 3).

**Table 3.** Changes in Blood Pressure, Medication, and Lifestyle Factors Among Participants With Hypertension

	Control group (n = 1133)			Intervention group (n = 2985)			Intervention effect^a^	
	No. (%)		Change from baseline (95% CI), %	No. (%)		Change from baseline (95% CI), %	OR (95% CI)	*P* value
	Baseline	12-Mo follow-up		Baseline	12-Mo follow-up			
SBP level, mean (SD), mm Hg	147.5 (18.0)	145.6 (18.0)	–1.9 (–3.3 to –0.5)	148.9 (18.5)	136.9 (13.4)	–12.0 (–12.8 to –11.1)	–10.1 (–11.7 to –8.5)	<.001
DBP level, mean (SD), mm Hg	85.5 (11.3)	83.3 (11.1)	–2.2 (–3.1 to –1.4)	85.1 (11.4)	81.1 (8.8)	–4.0 (–4.6 to –3.5)	–1.8 (–2.8 to –0.8)	<.001
BP classification								
Stage 1 hypertension	487 (43.0)	475 (41.9)	–1.1 (–5.0 to 2.9)	1167 (39.1)	811 (27.2)	−11.9 (−14.3 to −9.5)	0.90 (0.86 to 0.94)	<.001
Stage 2 hypertension	254 (22.4)	186 (16.4)	–6.0 (–9.0 to –3.0)	705 (23.6)	190 (6.4)	−17.3 (−19.1 to 15.4)	0.89 (0.86 to 0.93)	<.001
Stage 3 hypertension	68 (6.0)	67 (5.9)	–0.1 (–1.8 to 1.6)	215 (7.2)	34 (1.1)	−6.1 (−7.1 to −5.0)	0.94 (0.93 to 0.95)	<.001
Antihypertensive medication								
Yes	711 (62.8)	797 (70.3)	7.6 (3.8 to 11.4)	1926 (64.5)	2258 (75.6)	11.1 (8.8 to 13.5)	1.04 (0.99 to 1.08)	.08
No	422 (37.2)	336 (29.7)	–7.6 (–11.4 to –3.8)	1059 (35.5)	727 (24.4)	–11.1 (–13.5 to –8.8)	0.97 (0.92 to 1.01)	.12
No. of antihypertensive medications^b^								
1	479 (42.3)	289 (25.5)	–16.8 (–20.6 to –13.0)	1338 (44.8)	675 (22.6)	–22.2 (–24.6 to –19.9)	0.95 (0.91 to 0.99)	.02
2	191 (16.9)	323 (28.5)	11.7 (8.2 to 15.1)	472 (15.8)	911 (30.5)	14.7 (12.6 to 16.8)	1.03 (0.99 to 1.07)	.07
>2	41 (3.6)	170 (15.0)	11.4 (8.8 to 13.9)	116 (3.9)	617 (20.7)	16.8 (15.2 to 18.4)	1.06 (1.02 to 1.09)	.02
Lifestyle factors								
Current smoking	163 (14.4)	128 (15.0)	0.6 (–2.6 to 3.8)	470 (15.7)	419 (14.5)	–1.2 (–3.0 to 0.6)	0.98 (0.95 to 1.02)	.33
Current drinking	195 (17.2)	165 (19.3)	2.1 (–1.3 to 5.5)	541 (18.1)	463 (16.1)	–2.1 (–4.0 to –0.1)	0.96 (0.92 to 1.00)	.03
Overweight or obesity	825 (72.8)	784 (69.2)	–3.6 (–7.4 to 0.2)	2084 (69.8)	1955 (65.5)	–4.3 (–6.7 to –2.0)	0.99 (0.95 to 1.04)	.76
Regular exercise	478 (42.2)	481 (56.3)	14.1 (9.7 to 18.5)	1342 (45.0)	1717 (59.5)	14.6 (12.0 to 17.1)	1.00 (0.95 to 1.06)	.86

Abbreviations: DBP, diastolic blood pressure; OR, odds ratio; SBP, systolic blood pressure.

^a^ Data were presented as mean (SD) for continuous outcomes, and OR (95% CI) for dichotomous outcomes. Baseline variables were used for adjustment.

^b^ Numbers of medications were defined as follows: 0 for no drug use; 1 for 1 monotherapy; 2 for 2-drugs free combination or single-pill combination consisting of 2 active ingredients; >2 for more than 2-drugs free combination or single-pill combination consisting of more than 2 active ingredients.

After 12 months, the proportion of patients receiving antihypertensive medication in the intervention group was higher than in the control group (75.6% vs 70.3%), although the intervention effect between groups was insignificant (OR,1.04, 95% CI, 0.99-1.08; *P* = .08). Further analysis on the number of antihypertensive medications showed that the intervention effect on the use of monotherapy (OR, 0.95; 95% CI, 0.91-0.99; *P* = .02) and more than 2 medications (OR, 1.06; 95% CI, 1.02-1.09; *P* = .02) was significant. Throughout the study period, a total of 425 patients (337 in intervention group, 88 in control group) newly initiated antihypertensive medication (mainly monotherapy [74.6%]); more detailed information is provided in eTable 5 in Supplement 2. Compared with the control group, the change from baseline for lifestyle factors in the intervention group was not signficant: current smoking (OR, 0.98; 95% CI, 0.95-1.02; *P* = .33), overweight or obesity (OR, 0.99; 95% CI, 0.95-1.04; *P* = .76), or regular exercise (OR, 1.00; 95% CI, 0.95-1.06; *P* = .86) (Table 3). However, a substantial improvement in current drinking status compared with baseline was observed in the intervention group (OR, 0.96; 95% CI, 0.92-1.00; *P* = .03). The patterns of lifestyle factors change are shown in eFigure 4 in Supplement 2, and the data are presented in eTable 3 in Supplement 2.

No treatment-related serious adverse events occurred in either intervention or control groups. A total of 29 of 2985 patients (1.0%) in the intervention group experienced CVD events and 26 patients (0.9%) had drug-related adverse effects. In the control group, 13 of 1133 patients (1.1%) experienced CVD events, and 4 patients (0.3%) had medication-related adverse effects. Thirteen all-cause deaths (0.4%) occurred in the intervention group, and 6 (0.5%) occurred in the control group (eTable 6 in Supplement 2).

### Sensitivity Analysis

After multiple imputation for missing data, the difference in the reduction over 12 months was –9.7 mm Hg (95% CI, –11.4 to –8.1 mm Hg; *P* < .001) for SBP level and –1.7 mm Hg (95% CI, –2.7 to –0.7 mm Hg; *P* = .001) for DBP level (eTable 7 in Supplement 2). The difference in the increase in the proportion of patients with controlled hypertension was 7.6% (95% CI, 3.8%-11.3%; *P* < .001) in the control group and 24.0% (95% CI, 21.7%-26.3%; *P* < .001) in the intervention group; the intervention effect was still significant (OR, 1.18; 95% CI, 1.31-1.23; *P* < .001). Similar results were also observed in the BP classification and change of lifestyle factors.

## Discussion

In this multicenter randomized clinical trial, the multicomponent intervention delivered on a web-based platform resulted in better BP control in the intervention group compared with only usual care in the control group. The intervention lowered BP levels, increased use of antihypertensive medications, and improved some health-related lifestyle factors.

Previous studies have observed that community health worker–led^24^ or BP telemonitoring–based^25^ interventions increased the BP control rate by 16.0% to 20.6%. In the present study, the intervention effect on BP control was 24.6% (OR, 1.18; 95% CI, 1.13-1.24; *P* < .001). The BP change difference between the groups was –10.1 mm Hg for SBP level and –1.8 mm Hg for DBP level, which was greater than the change reported in previous studies based on WeChat,^26^ text messaging,^27^ and community primary care physicians.^28^ Although it was undeniable that there were variations in design, intervention, and time frame among different studies, which might lead to different results, the multicomponent intervention delivered on a web-based platform in the present study was effective in improving BP control.

The current intervention led to significant changes in BP classification and lifestyle factor of drinking, although the proportion of smoking, regular exercise, and overweight or obesity were not significantly changed between groups. Findings on changes in lifestyle factors in the present trial were similar to those of previous studies based on text messaging,^15,29^ which reported no significant changes in physical activity, BMI, or smoking status. According to previous studies conducted in rural areas^30^ and workplaces,^9^ after 2 years of intervention, changes in the lifestyle factors of BMI and smoking were not significant. The findings indicated that improvements in behavior occurred within the first few months and plateaued due to the enthusiasm for lifestyle changes waning over time, which could be the reason why no significant changes were observed in BMI group, smoking, and regular exercise between groups.

In the present study, the possible mechanism for the intervention effect on BP control rate may be the coordination between various intervention components, which served to enhance the communication and exchange between patients and primary care physicians. First, the web-based intervention, which was an improvement over traditional health methods, brought disparate interventions together. Automatic BP classification and follow-up plan reminders could help primary care physicians treat patients with hypertension more efficiently. Second, the continued health education about CVD prevention and lifestyle modification may increase patients’ health literacy, which was associated with better BP control.^31^ Patients who had higher health literacy were more likely to have controlled BP. Third, regular follow-up motivated the adherence to a healthy lifestyle and drug treatment, which was effective in improving BP control. Besides, the Hawthorne effect could also have altered BP control because the follow-up period was short (12 months). Although the intervention effects of lifestyle factors and antihypertensive medication were insignificant between groups, further analysis showed a lower proportion of monotherapy use and higher proportion of combination medication use in the intervention group than in the control group after 12 months of intervention, which might be the main reason for the significant intervention effect. Moreover, among the patients receiving drug treatment, the intervention effect in the intervention group was also significantly greater than in the control group.

In recent years, telemedicine has become more important in the management of hypertension due to its potential benefits over traditional methods^32^; especially during the COVID-19 pandemic, restrictions on travel limited the accessibility of care and medications. Text message– or application-based interventions supporting health management for people with or at risk for cardiovascular disease have been used in many studies, such as the TEXT ME (Tobacco, Exercise and Diet Messages) trial,^27^ CHAT (Cardiovascular Health and Texting Messaging) study,^15^ and REMOTE-CR (Remotely Monitored Exercise-based Cardiac Telerehabilitation) trial.^33^ To date, however, there are few studies that have used a theory-based platform between patients and health professionals.

To our knowledge, the present randomized clinical trial was the largest to test the validity of a web-based hypertension management intervention and helped address the gap in evidence to show that such a platform was effective. As the most important part of chronic disease management in China, primary care physicians in community health care centers face a great management burden. Such a web-based intervention provided these physicians with a simple, convenient, and continual means of disease management. It not only reduced the management burden but also promoted the accessibility of quality medical resources.

### Limitations

Some limitations to the present study need to be considered. First, the follow-up period was relatively short (only 12 months), and we had no idea whether the BP reduction and lifestyle improvement would continue to the future. The difference from baseline to 12 months could be an indication of sustained change in the future. Second, the evaluation of lifestyle change was based on patients’ self-reported data, making recall bias unavoidable. Third, almost 11% of patients in both groups were lost to follow-up during the intervention. However, the baseline characteristic differences between patients who completed follow-up and those who were lost to follow-up were not significant. The power analysis indicated that there was still a power of 89% (1% decrease from baseline) under the present sample size to detect a significant difference.

Fourth, patients who were randomized to the control group received no regular follow-up; thus, we could not analyze the intervention effect between the intervention and control groups during the intervention period. However, all patients in the control group received regular management from the local community primary care physicians under Essential Public Health Service, including health education and promotion. Health management for people with hypertension was implemented in 2009 in China. Therefore, we believe the multicomponent intervention delivered on a web-based platform was more effective than the usual care. Fifth, due to the lack of relevant data, we could not assess the primary care physicians’ satisfaction with this intervention and to what extent the primary care physicians engaged patients in self-managing their BP; this situation should be improved in future studies. We did not consider excessive alcohol and illegal substance consumption in the exclusion criteria. Given the multicomponent nature of the intervention, it did not seem possible to determine the effect of any specific component.

## Conclusions

In this cluster randomized clinical trial, patients in the intervention group who received a multicomponent intervention delivered on a web-based platform achieved greater improvement in BP control rate compared with patients in the control group, who received only usual care. The study provided clinical evidence that telemedicine may be an effective way to manage patients with hypertension in the community and may generate public health benefits across diverse populations.

## Notes

Supplement 1.
Trial Protocol

Supplement 2. **eTable 1.** Baseline Characteristics for All Participants Enrolled in the Study **eTable 2.** Comparison of Follow-up and Lost to Follow-up Patients **eTable 3.** Changes From Baseline for BP Control Rate, BP Levels and Lifestyle Factors **eTable 4.** Hypertension Control at Baseline and Follow-up **eTable 5.** Newly Initiated Antihypertensive Medication **eTable 6.** Adverse Events in the Study **eTable 7.** Sensitivity Analysis for Primary and Secondary Outcomes After Imputing the Missing Data in the Study **eFigure 1.** CONSORT Flow Diagram **eFigure 2.** Overview of the Web-Based Platform **eFigure 3.** Subgroup Analysis for Change in Blood Pressure at 12 Months **eFigure 4.** Mean Change in Lifestyle Factors Over Time

Supplement 3.
Data Sharing Statement
