# Supplementary material for: Effect of a Multicomponent Intervention Delivered on a Web-Based Platform on Hypertension Control: A Cluster Randomized Clinical Trial
Source: JAMA Netw Open. 2022 Dec 7;5(12):e2245439. doi: 10.1001/jamanetworkopen.2022.45439 (PMC9856259; doi:10.1001/jamanetworkopen.2022.45439)
Supplement: Supplement 1. — Trial Protocol [file jamanetwopen-e2245439-s001.pdf]

Original trial protocol and statistical analysis plan

Study title: Cardiovascular risk assessment and early intervention studies based on environmental and epigenetic inheritance

Study Number: 2017-I2M-1-004

Funding Support: Projects in Chinese Academy of Medical Science Innovation Project

Funding Organization: Chinese Academy of Medical Science

Trial Registration: ChiCTR1800017791

Original Protocol: Version 1.0 1 December 2017

Confidentiality Statement: The information contained herein is confidential and the proprietary property of Division of Prevention and Community Health, National Center for Cardiovascular Disease, China and any unauthorized use or disclosure of such information without the prior authorization of Division of Prevision and Community Health, National Center For Cardiovascular Disease, China is expressly prohibited.

Introduction

Cardiovascular disease (CVD) has become a major disease threatening the health of residents across the world. It was estimated that about 3.5 million people die of cardiovascular disease every year, meaning that 1 person dies of cardiovascular disease every 10 seconds, which brings a huge economic burden to China. Raised blood pressure (BP) is established as a strong independent risk factor for CVD, accounting for 70% of strokes and 50% of myocardial infarction in China. However, the rate of awareness, treatment, and control in China is still in a low level.

According to previous works, traditional interventions concerning health education, risk factors control, as well as lifestyle modification have succeeded in lowing the BP level and improving the control rate of hypertension patients, no matter in community or work places. But compared with western countries, the blood pressure control rate in China is still not unsatisfactory. The lack of hypertension health education resources in China leads to the lack of hypertension health knowledge among high-risk groups. Moreover, many problems exposed in the traditional outpatient follow-up mode of hypertension patients often lead to the low frequency of blood pressure monitoring, the unguaranteed compliance of patients, and the inability to exclude "white coat hypertension". The collection, analysis and management of patients' risk factor information, doctors' adjustment of medication and patients' feedback are often separated.

In recent years, with the development of the internet and mobile technology, information collection and automatic management through wearable devices could provide patients with health knowledge timely, reminded of medication, and help them pay more attention to their own conditions, adhere to the improvement of lifestyle, and strengthen self-management. Besides, remote dynamic monitoring can also be performed, which helps doctors to grasp the blood pressure fluctuations of

patients in real time, reducing the burden of medical staff; at the same time, high-quality expert resources can be used for training, consultation and guidance through remote video technology on the platform, which could improve the technical level of doctors effectively. Therefore, we intend to establish a web-based telemedicine platform for hypertension patients based on the standardized management guideline of hypertension; and evaluate the effect of this platform in the management of hypertension. Herein, we hypothesize that the web-based platform could improve BP control in communities across China, and a cluster-controlled trial will be conducted to test this hypothesis.

Primary Objective:

To build and improve the web-based management platform for hypertension and coronary heart disease, and assess the changes in the percentage of patients with controlled hypertension from baseline to the 12-month follow-up.

Secondary Objective:

1. Assessed the changes of BP level from baseline to the 12-month follow-up;
2. Assessed the changes of lifestyle factors concerning smoking, drinking, regular exercise and overweight/obesity, from baseline to 12-month follow-up.

Study Design:

The study is designed to a multicenter, randomized, controlled trial conducted to test the effect of the web-based platform on improving hypertension control rate in the community.

For this program, a coordinating center will be established at Fuwai Hospital, Beijing. Then, 22 urban medical institutions will be selected as sub-centers,

representing various geographic locations in China. The criteria for selecting sub-centers are as follows: (1) district or county-level medical institutions that are interested in this project, willing to actively participate, and able to complete tasks as required; (2) being able to coordinate with community health service centers and promote project progress; (3) having ability to organize relevant training, supervision, and website Q&A activities according to the requirements of the project plan; (4) having good organization and coordination ability; (5) promising to participate in the program for a minimum of 1 years. Within each sub-center, 2 community health centers will be assigned to the intervention group, and 1 community health center assigned to the control group. To minimize the potential imbalance between groups, community health center assigned to the intervention and control group within each sub-center, should be matched by medical level, population size, and level of economic development. Within each community health center, at least 67 potential participants will be selected and invited for baseline assessments, and the patients will be allocated to intervention or usual care.

For patients randomized to the intervention group, the web-based platform was used to conduct health intervention and there will be monthly follow-up visits within 1 year duration. Patients randomized to the control group just receive usual care consisted of existing Essential Public Health Service.

Study Duration:

Subjects will receive intervention for a minimum of 12 months.

Inclusion Criteria:

1. Essential hypertension.

2. Subjects with high coronary heart disease risk (the following two items must be met simultaneously)

   (1) at least with hypercholesterolemia or (and) diabetes

   (2) Additionally with two or more risk factors as following: overweight and obese; smoking; lack of activity; male or postmenopausal women; family history of cardiovascular disease.

3. Local resident population;

4. Signed consent form and agreed to receive 12-month intervention;

5. Promised not to drop out due to the long term leave, going abroad or other reason during the next one year.

Exclusion Criteria:

1. Secondary hypertension, hypertension emergency

2. Type 1 diabetes;

3. Type 2 diabetes mellitus with acute metabolic disorder;

4. Secondary dyslipidemia patients (secondary to patients with type 2 diabetes not included);

5. Acute myocardial infarction or stroke within 3 months;

6. Patients with chronic kidney disease (eGFR <60ml/min/1.73m2 or urine albumin/creatinine ratio⩾30);

7. Pregnant women and nursing mothers;

8. Difficult to cooperate (psychiatric illness, hearing difficulty, or physical incapacitation);

9. With serious diseases, and life expectancy is less than one year;

10. Healthcare providers.

Intervention Strategy:

The intervention will last 12 months, which included the following components: 1. primary prevention program for cardiovascular disease; 2. a standardize management protocol for hypertension based the current guideline; 3. a web-based platform were established to support the implementation of interventions.

1. Primary prevention program for cardiovascular disease

In order to improving the health awareness and empower patients with hypertension manage cardiovascular disease risk factors, a comprehensive primary prevention program for cardiovascular disease will be implemented. According to the Chinese guideline for the Management of Hypertension, the program including the following components: (1) health education. The officers will provide general knowledge of the cardiovascular disease in the way of text messages. The topics including prevalence, risk factors, prevention and management of CVD. (2) Reasonable diet. Nutrition education, and healthy diets information was provided to the patients. Patients were educated to restrict total calories intake, choose a low-salt, low-fat diet, maintain balanced nutrition, and increase the intake of fruits and vegetables. (3) Physical activity. Participants were encouraged to increasing physical activity, the type of activity is mainly aerobic exercise (heart rate = 170-age), and the frequency of activity is recommended to be at least 3 times a week, at least 30 minutes each time. (4) Weight loss. Setting goals for weight control, the ideal goal was

BMI<24Kg/m^2, waist circumference<90cm for male, <85cm for female. (5) Tobacco cessation. Educating patients about the dangers of smoking, and developing a smoking cessation plan with them. Informing the patient's family and friends to help them quit smoking. (6) Alcohol cessation. Patients were advised not to drink alcohol, if drinking, in small amounts. Drinking no more than two times a week. (7) Self-monitoring. Patients were encouraged to take self-monitoring at home.

2. Clinic Component

Patients with hypertension will be managed for at least 1 years by physician in clinic trained by a standardized BP management protocol. The protocol, which is based on Chinese Guidelines for the Management of Hypertension, include the following: classification of hypertension, stratification of global risk, targets for BP control, the principles of treatments, lifestyle changes, and follow-up management. BP control targets are as follows: <140/90 mmHg for uncomplicated hypertension; <130/80 mmHg for patients with diabetes, cerebrovascular disease, stable coronary heart disease, or chronic kidney failure. The principles of treatment are based on patients' risk stratification (Figure). The physicians should make oral health education on lifestyle changes and prescribe the drugs according to patients' conditions. The patients will be instructed to visit the designated clinics monthly until the end of this trail, during which the measurement of BP as well as the adjustment of individual therapeutic regimen made. The project officers should be responsible for supervising and assisting hypertensive patients in regular follow-up. Moreover, patients will be referred to a higher-level hospital when necessary, and then return to the designated clinics for follow-up when they are in a stable condition.

3. A web-based platform

A web-based management platform oriented by 2017 Chinese hypertension management guidelines was established by National Center for Cardiovascular Diseases to facilitate the implementation of interventions. The main functions of the platform include establishing digital medical records for patients, helping doctors achieve one-to-one management, guiding patients to conduct self-management, carrying out health follow-up intervention (condition warning, health advice, medication reminder and health education, etc.), and providing doctors with auxiliary decision-making. (1) Data collection: health providers logged into the management platform and established electronic records based on the basic information, medical history, family history of cardiovascular disease, lifestyle factors, physical examination and current medication collected in the baseline survey. (2) Follow-up management: after the digital record was established, the platform would evaluate the data automatically and a personalized follow-up schedule according to the hypertension guideline would be generated. The platform could also alert the health providers as the follow-up date nears, for those with poor blood pressure control, the platform will highlight the label to remind doctors to carry out timely follow-up to avoid the occurrence of adverse events. During each visit the compliance of medicine therapy, lifestyle changes, BP level, and adverse events would be evaluated and entered into the platform. (3) Cardiovascular disease risk evaluation: based on the input patient information, the risk assessment of patients is automatically carried out, and health providers could provide personalized health education based on the results of risk assessment, help patients carry out self-management, adhere to lifestyle adjustment, control the risk factors related to hypertension, and prevent the occurrence of cardiovascular events.

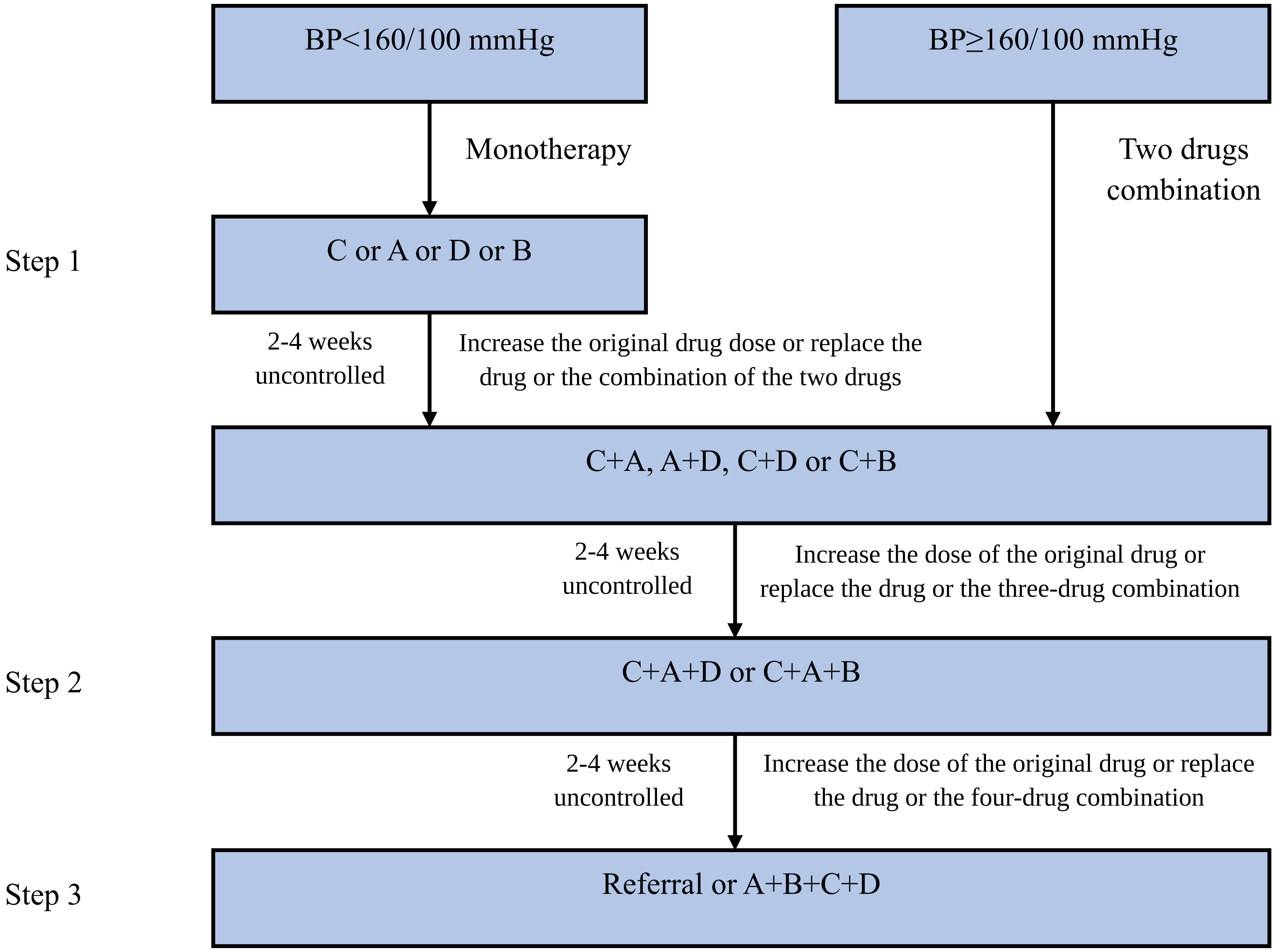

Figure. Hypertension Medication Management Algorithm in Intervention Group

Abbreviations: A, angiotensin converting enzyme inhibitor, or angiotensin receptor antagonist; B, beta blocker; C, calcium channel blocker; D, diuretic.

Effectiveness Assessments:

There will be two examinations for both groups, at baseline and 12 months (after 1 year intervention). Indicators including BP control rate, BP levels, BMI and lifestyle factors will be assessed.

All outcome assessments will be conducted at baseline and at 12-month follow-up in exactly the same way in the clinics for all patients, irrespective of their assignment to intervention or control group. For the intervention patients, monthly follow-up visits will be made within 1 year duration to document the BP and lifestyle factors.

Statistical Method:

All patients who completed the 12 months follow-up were included in the analysis for the primary outcome and secondary outcome. The baseline differences between patients included and unincluded, patients in the intervention group and those in the control group, will be examined by mixed-effects model. The intervention effect will be performed as the difference between two groups, it will be calculated as intervention (values at 12 months minus values at baseline) minus control (values at 12 months minus values at baseline), and evaluated by mixed-effects models (PROC MIXED for continuous variables, PROC GLIMMIX for categorical variables), which included a random cluster effect (community health centers). The subgroup analyses of primary outcome will be conducted to assess the intervention effect on subgroups of sex, age group, educational attainment, region, BMI group and CVD family history. To evaluate the robustness of the results of the primary analyses, multiple-imputation method was adopted to handle missing data on outcome and covariates in a sensitivity analysis.

The results will be summarized as rate (%), mean, standard deviation (SD), standard error (SE), and 95% confidence interval (CI) when appropriate. All data analyses will be conducted using SAS 9.4 (SAS institute, Cary, NC, USA) and a two-sided P-value <0.05 will be considered statistically significant.

Sample Size Consideration:

The study is designed as an effectiveness trial of active interventions, compared with the usual care group. Considering the intervention benefits and ethical issues, the study was designed to recruit patients using a 2:1 ratio. Cluster randomization power analysis was performed for the two independent proportions, and with 2.5% type I error and intra-cluster correlation coefficient (ICC) of 0.01, we estimated that a sample size of 3,000 intervention patients and 1,500 control patients would provide 90% power to detect an improvement of 20% in BP control between the two groups and allowing 20% loss to follow-up in each group. To ensure an enough power, 3,350 intervention patients and 1,675 control patients were recruited in this study.

Reference:

1. Roth GA, Johnson C, Abajobir A, et al. Global, Regional, and National Burden of Cardiovascular Diseases for 10 Causes, 1990 to 2015. Journal of the American College of Cardiology 2017; 70(1): 1-25.
2. Chen W, Gao R, Liu L, et al. Report on cardiovascular disease in chine 2013. Chinese Circulation Journal. 2014: 29(07): 487-91.
3. Lawes CM, Rodgers A, Bennett DA, et al. Blood pressure and cardiovascular disease in the Asia Pacific region. Journal of hypertension 2003; 21(4): 707-16.
4. Thomopoulos C, Parati G, Zanchetti A. Effects of blood pressure lowering on outcome incidence in hypertension. 1. Overview, meta-analyses, and meta-regression analyses of randomized trials. Journal of hypertension 2014; 32(12): 2285-95.
5. Wang Z, Hao G, Wang X, et al. Short-term hypertension management in community is associated with long-term risk of stroke and total death in China: A community controlled trial. Medicine (Baltimore) 2016; 95(48): e5245.
6. Li G, Zhang P, Wang J, et al. Cardiovascular mortality, all-cause mortality, and diabetes incidence after lifestyle intervention for people with impaired glucose tolerance in the Da Qing Diabetes Prevention Study: a 23-year follow-up study. Lancet Diabetes Endocrinol 2014; 2(6): 474-80.
7. Chen J, Wu X, Gu D. Hypertension and cardiovascular diseases intervention in the capital steel and iron company and Beijing Fangshan community. Obes Rev 2008; 9 Suppl 1: 142-5.
8. Horigan G, Davies M, Findlay-White F, Chaney D, Coates V. Reasons why patients referred to diabetes education programmes choose not to attend: a systematic review. *Diabet Med* 2017; **34**(1): 14-26.
9. Watson AJ, Singh K, Myint UK, et al. Evaluating a web-based self-management program for employees with hypertension and prehypertension: a randomized clinical trial. *Am Heart J* 2012; **164**(4): 625-31.
10. Omboni S, Gazzola T, Carabelli G, Parati G. Clinical usefulness and cost effectiveness of home blood pressure telemonitoring: meta-analysis of randomized controlled studies. *Journal of*

hypertension 2013; **31**(3): 455-67; discussion 67-8.
11. Chow CK, Redfern J, Hillis GS, et al. Effect of Lifestyle-Focused Text Messaging on Risk Factor Modification in Patients With Coronary Heart Disease: A Randomized Clinical Trial. *JAMA* 2015; **314**(12): 1255-63.

Appendix A: Informed Consent

You are participating in a web-based intervention program for hypertension and high coronary heart disease risk. The program is organized by Fuwai Hospital, Peking Union Medical College & Chinese Academy of Medial Science (National Center for Cardiovascular Disease, China), and conducted by the medical institutions in your local community. The program will train the community doctors, who will conduct targeted interventions (including health education and lifestyle guidance) for high-risk individuals through the web-based platform, standardized treatment and follow-up management for patients to facilitate the control of blood pressure, plasma glucose, as well as plasma lipids, and prevent cardiovascular disease.

If you are to be participated in this program, for the subsequent 1 years, the project officer in your community will be responsible for guidance of your lifestyle modification, and conduct standardized treatment and follow-up for your hypertension, diabetes, and dyslipidemia. During the follow-up management, you need to cooperate in the follow aspects: participate in the health promotion activities; check behavior change, medication reminders, and get health counseling and guidance regularly; visit a designated doctor regularly, so that the same doctor can observe the changes in the condition and the effect of prevention and treatment for a long time; the doctor is obligated to explain the hypertension, diabetes, and dyslipidemia related knowledge and answer you question during your follow-up visit. According to the requirement of the program, fasting blood glucose, blood lipids (total cholesterol, triglycerides, and high-density lipoprotein cholesterol), uric acid, creatinine, glutamic-pyruvate and glutamic-oxalacetic transaminase, electrolytes, creatine kinase, urine routine tests, ECG and carotid ultrasound were performed at

the beginning of the program and 1 year latter at the end of this program, all of these detections are free of charge. The drugs that the doctor choose for you in the course of treatment are all conventional drugs, and there are no special medication requirements in this program.

Participation in the program is voluntary. You have right to withdraw from this activity at any time.

Before signing this consent form, I have read or others have read to me, and I have the opportunity inquire and receive a satisfactory reply. I will volunteer for this program. After participating in the program, I am willing to accept the follow-up management and guidance of doctors in designated hospitals.

Subjects with signature__________________ __________________Date

Appendix B: Questionnaire (For baseline and 12 month)

Subcenter ID □□ No. of community □□ No. of patients □□□□

Identity number: □□□□□□□□□□□□□□□□□□

Date of filling: Year □□□□ Month □□ Date □□

Section A: Basic information

A1. Name: ____________

A2: Gender: 1= Male 2=Female

A3: Race: ____________

A4: Date of birth: Year □□□□ Month □□ Date □□

A5: Educational attainment:

1=Postgraduate 2=Bachelor 3=High school 4=Middle school and blow

A6: Number of family: □□

A7: Marital status:

0=Unmarried 1=Married 2=Divorced 3=Widowed

A8: Employment status:

0=On the job 1=Retired 2=Student 3=Unemployed

A8: Are you a local population? 0= No 1=Yes

A9: How long have you lived here?

1=≤6 months 2=7-12 months 3=12-36 months 4=≥36 months

Section B: Health Status (Determined according to the current Chinese clinical guidelines)

B1: Do you have hypertension? 0=No 1=Yes 9=Unclear

If yes, time of diagnosis: Year □□□□ Month □□, duration: □□ years.

Current BP levels (SBP/DBP): □□□/□□□ mmHg

B2: Do you have dyslipidemia? 0=No 1=Yes 9=Unclear

If yes, time of diagnosis: Year □□□□ Month □□, duration: □□ years.

B3: Do you have diabetes? 0=No 1=Yes 9=Unclear

If yes, time of diagnosis: Year □□□□ Month □□, duration: □□ years.

B4: Do you have heart disease? 0=No 1=Yes 9=Unclear

If yes, please select:

0= coronary heart disease 1= angina pectoris 2= myocardial infarction

3= Coronary artery reconstruction 4=Heart failure

Time of first onset: Year □□□□ Month □□, number of onset □□

B5: Do you have cerebrovascular disease? 0=No 1=Yes 9=Unclear

If yes, please select: 0= Ischemic stroke 1= Cerebral hemorrhage

Time of first onset: Year □□□□ Month □□, number of onset □□

B6: Do you have chronic kidney disease? 0=No 1=Yes 9=Unclear

If yes, time of diagnosis: Year ☐☐☐☐ Month ☐☐, duration: ☐☐ years.

Section C: Family History (Positive is defined as parents/siblings suffer from the disease before the age of 50 years)

C1: Hypertension ☐No ☐Yes ☐Unclear

If yes, ☐Father ☐Mother

C2: Dyslipidemia ☐No ☐Yes ☐Unclear

If yes, ☐Father ☐Mother

C3: Diabetes ☐No ☐Yes ☐Unclear

If yes, ☐Father ☐Mother

C4: Coronary heart disease ☐No ☐Yes ☐Unclear

If yes, ☐Father ☐Mother

C5: Stroke ☐No ☐Yes ☐Unclear

If yes, ☐Father ☐Mother

Section D: Lifestyle Behaviors

D1: Do you restrict salt intake? 0=No 1=Yes

D2: Do you restrict fat intake? 0=No 1=Yes

D3: Do you exercise? 0=No 1=Yes

If yes, ☐☐days per we ☐☐☐minutes per day

D4: Do you smoking currently (at least one cigarette per day)? 0=No 1=Yes

If yes, how many cigarettes do you smoke daily on average? □□

D5: Do you drinking currently (at least once per week)? 0=No 1=Yes

If yes, how many times do you drink per week on average? □□

D6: Do you perceive depressive, stress, nervous, anxious or angry? 0=No 1=Yes

Section E: Physical Examination

E1: Height □□□.□ cm; Weight □□□.□ Kg

E2: Waist circumference □□□.□ cm; Hip circumference □□□.□ cm

E3: Body fat percentage □□□.□; Visceral fat index: □□□.□;

Basal metabolic rate □□□□

E4: Blood pressure (SBP/DBP)

The first time □□□/□□□mmHg; the second time □□□/□□□mmHg

E5: Heart rate: □□□ per minute

Section F: Biochemical test

F1: Fasting glucose □□.□□mmol/L or □□□mg/dL

F2: Triglycerides □□.□□mmol/L or □□□mg/dL

F3: Total cholesterol □□.□□mmol/L or □□□mg/dL

F4: High density lipoprotein cholesterol □□.□□mmol/L or □□□mg/dL

F5: Serum creatinine □□.□□mmol/L or □□.□□mg/dL

F6: Alanine aminotransferase □□□□U/L

Aspartate aminotransferase □□□□U/L

F7: Serum creatine kinase □□□.□U/L mmol/L

F8: Uric acid □□□□.□μmmol/L or □□.□□mg/dL

F9: Urine protein 0="-" 1="+" 2="++" 3="+++ and above"

F10: Blood electrolyte

Serum sodium □□□mmol/L potassium □□.□mmol/L

chlorine □□□mmol/L

Appendix C: Questionnaire (For monthly follow-up in intervention group)

Subcenter ID □□ No. of community □□ No. of patients □□□□

Date of filling: Year □□□□ Month □□ Date □□

Section A: Physical Examination

A1: Weight □□□.□ Kg

A2: Waist circumference □□□.□ cm; Hip circumference □□□.□ cm

A3: Body fat percentage □□□.□; Visceral fat index: □□□.□;

Basal metabolic rate □□□□

A4: Blood pressure (SBP/DBP)

The first time □□□/□□□mmHg; the second time □□□/□□□mmHg

A5: Heart rate: □□□ per minute

Section B: Biochemical test

B1: Fasting glucose □□.□□mmol/L or □□□mg/dL

B2: Triglycerides □□.□□mmol/L or □□□mg/dL

B3: Total cholesterol □□.□□mmol/L or □□□mg/dL

B4: High density lipoprotein cholesterol □□.□□mmol/L or □□□mg/dL

Section C: Lifestyle Behaviors

C1: Do you restrict salt intake? 0=No 1=Yes

C2: Do you restrict fat intake? 0=No 1=Yes

C3: Do you exercise? 0=No 1=Yes

If yes, ☐☐days per we ☐☐☐minutes per day

C4: Do you smoking during the follow-up period (at least one cigarette per day)?

0=No 1=Yes

If yes, how many cigarettes do you smoke daily on average? ☐☐

C5: Do you drinking during the follow-up period (at least once per week)?

0=No 1=Yes

If yes, how many times do you drink per week on average? ☐☐

C6: Do you perceive depressive, stress, nervous, anxious or angry? 0=No 1=Yes
