# Supplementary material for: Effect of a Multicomponent Intervention Delivered on a Web-Based Platform on Hypertension Control: A Cluster Randomized Clinical Trial
Source: JAMA Netw Open. 2022 Dec 7;5(12):e2245439. doi: 10.1001/jamanetworkopen.2022.45439 (PMC9856259; doi:10.1001/jamanetworkopen.2022.45439)
Supplement: Supplement 2. — eTable 1. Baseline Characteristics for All Participants Enrolled in the Study eTable 2. Comparison of Follow-up and Lost to Follow-up Patients eTable 3. Changes From Baseline for BP Control Rate, BP Levels and Lifestyle Factors eTable 4. Hypertension Control at Baseline and Follow-up eTable 5. Newly Initiated Antihypertensive Medication eTable 6. Adverse Events in the Study eTable 7. Sensitivity Analysis for Primary and Secondary Outcomes After Imputing the Missing Data in the Study eFigure 1. CONSORT Flow Diagram eFigure 2. Overview of the Web-Based Platform eFigure 3. Subgroup Analysis for Change in Blood Pressure at 12 Months eFigure 4. Mean Change in Lifestyle Factors Over Time [file jamanetwopen-e2245439-s002.pdf]

Supplemental Online Content

Zhou H, Wang X, Yang Y, et al. Effect of a multicomponent intervention delivered on a web-based platform on hypertension control: a cluster randomized clinical trial. *JAMA Netw Open*. 2022;5(12):e2245439. doi:10.1001/jamanetworkopen.2022.45439

eTable 1. Baseline Characteristics for All Participants Enrolled in the Study

eTable 2. Comparison of Follow-up and Lost to Follow-up Patients

eTable 3. Changes From Baseline for BP Control Rate, BP Levels and Lifestyle Factors

eTable 4. Hypertension Control at Baseline and Follow-up

eTable 5. Newly Initiated Antihypertensive Medication

eTable 6. Adverse Events in the Study

eTable 7. Sensitivity Analysis for Primary and Secondary Outcomes After Imputing the Missing Data in the Study

eFigure 1. CONSORT Flow Diagram

eFigure 2. Overview of the Web-Based Platform

eFigure 3. Subgroup Analysis for Change in Blood Pressure at 12 Months

eFigure 4. Mean Change in Lifestyle Factors Over Time

This supplemental material has been provided by the authors to give readers additional information about their work.

eTable 1. Baseline Characteristics for All Participants Enrolled in the Study				
	**Total (4,709)**	**Control (1,570)**	**Intervention (3,139)**	***P value***
Age, mean(SD), y	61.5(9.4)	61.2(9.7)	61.5(9.4)	.52
Sex, male, No.(%)	2,136(45.4)	747(47.6)	1,389(44.2)	.03
smoking, No.(%)	716(15.2)	226(14.4)	490(15.6)	.28
drinking, No.(%)	841(17.9)	279(17.8)	562(17.9)	.91
BMI group, No.(%)				.24
normal	1,316(28.0)	409(26.1)	907(28.9)	
overweight	2,052(43.6)	699(44.5)	1,353(43.1)	
obesity	1,260(26.8)	434(27.6)	826(26.3)	
SBP, mean(SD), mmHg	148.6(18.5)	147.5(17.7)	148.6(18.5)	.12
DBP, mean(SD), mmHg	85.0(11.4)	85.8(11.5)	85.0(11.4)	.002
BP control, No.(%)	1430(30.4)	358(22.8)	753(24.0)	.37
CVD family history				.79
no	2,891(61.4)	968(61.7)	1,923(61.3)	
yes	1,818(38.6)	602(38.3)	1,216(38.7)	
Antihypertensive medication	3,007(63.9)	991(63.1)	2,016(64.2)	.41
Region, No.(%)				.24
west	1,331(28.3)	450(28.7)	881(28.1)	
central	1,140(24.2)	399(25.4)	741(23.6)	
east	2,238(47.5)	721(45.9)	1517(48.3)	
Abbreviations: BMI, Body Mass Index; SBP, systolic Blood Pressure; CVD, cardiovascular disease; SD, standard deviations.				

© 2022 Zhou H et al. *JAMA Network Open.*

eTable 2. Comparison of Follow-up and Lost to Follow-up Patients			
	**Follow-up (4,198)**	**Lost of follow-up (511)**	***P* value**
Age group, No.(%)			.13
<65	2,457(58.5)	317(62.0)	
≥65	1,741(41.5)	194(38.0	
Sex, No.(%)			.15
Male	1,889(45.0)	247(48.3)	
Female	2,309(55.0)	264(51.7)	
BMI mean(SD), Kg/m^2	26.0(3.5)	26.1(3.3)	.66
BP mean(SD), mm Hg			
SBP	148.4(18.4)	146.9(17.3)	.13
DBP	85.2(11.4)	85.8(11.9)	.18
BP control, No.(%)	978(23.3)	133(26.0)	.17
Antihypertensive medication, No. (%)	2,684(63.9)	323(63.2)	.64

Abbreviations: BMI, body mass index; BP, blood pressure; SBP, systolic blood pressure; DBP, diastolic blood pressure; SD, standard deviations; CVD, cardiovascular disease.

© 2022 Zhou H et al. *JAMA Network Open.*

eTable 3. Changes from baseline for BP control rate, BP levels and Lifestyle factors					
	Group	Months			
		3	6	9	12
BP control rate (%,95%CI)	intervention	20.9(18.7-23.2)	27.3(25.1-29.4)	30.6(28.3-32.8)	24.6(22.3-26.9)
	control	-	-	-	7.7(3.9-11.4)
SBP (mm, 95%CI)	intervention	-10.7(-11.5 to -9.9)	-13.6(-14.4 to -12.8)	-14.1(-14.9 to -13.3)	-12.0(-12.8 to -11.1)
	control	-	-	-	-1.9(-3.3 to -0.5)
DBP (mm, 95%CI)	intervention	-2.8(-3.3 to -2.3)	-4.4(-4.9 to -3.9)	-4.8(-5.3 to -4.3)	-4.0(-4.6 to -3.5)
	control	-	-	-	-2.2(-3.1 to -1.4)
regular exercise (%,95%CI)	intervention	12.2(9.8-14.7)	13.5(11.1-15.9)	15.4(13.0-17.8)	14.4(11.9-16.9)
	control	-	-	-	14.1(9.8-18.5)
Smoking (%,95%CI)	intervention	0.6(-1.2-2.4)	-1.8(-3.6 to -0.1)	-2.6(-4.3 to -0.9)	-1.2(-3.0 to 0.6)
	control	-	-	-	0.4(-2.7 to 3.6)
Drinking (%,95%CI)	intervention	0.7(-1.2 - 2.7)	-0.2(-2.1 - 1.6)	-2.2(-4.1 to -0.4)	-2.1(-4.0 to -0.2)
	control	-	-	-	2.1(-1.3 to 5.5)
Obesity (%,95%CI)	intervention	-4.8(-7.1 to -2.6)	-7.6(-9.8 to -5.3)	-8.0(-10.3 to -5.8)	-5.3(-7.7 to -3.0)
	control	-	-	-	-4.4(-8.2 to -0.5)

Abbreviations: BP, blood pressure

© 2022 Zhou H et al. *JAMA Network Open.*

eTable 4. Hypertension Control at Baseline and Follow-up

Baseline	12 months	Control Group (1,133)	Intervention Group (2,985)	Intervention Effect	*P value*
uncontrolled	uncontrolled	679(59.9)	1425(47.7)	-	
uncontrolled	controlled	199(17.6)	879(29.5)	1.17(1.13-1.21)	<.001
controlled	uncontrolled	112(9.9)	145(4.9)	-	
controlled	controlled	143(12.6)	536(18.0)	-	

Data were presented as NO.(%).

© 2022 Zhou H et al. *JAMA Network Open.*

eTable 5. Newly Initiated Antihypertensive Medication				
	**Total (425)**	**Control (88)**	**Intervention (337)**	***P* value**
Number of antihypertensive medication*, No.(%)				.15
1	317(74.6)	68(77.3)	249(73.9)	
2	94(22.1)	20(22.7)	74(22.0)	
>2	14(3.3)	0	14(4.2)	
Drug Type for Monotherapy, No.(%)				.04
ACEI	71(22.4)	15(22.1)	56(22.5)	
ARB	68(21.5)	11(16.2)	57(22.9)	
CCB	144(45.4)	31(45.6)	113(45.4)	
β-blockers	4(1.3)	3(4.4)	1(0.4)	
Diuretics	21(6.6)	5(7.4)	16(6.4)	
other	9(2.8)	3(4.4)	6(2.4)	

Abbreviations: ACEI, angiotensin converting enzyme inhibitor; ARB, angiotensin receptor antagonist; CCB, calcium channel blocker; Other, traditional Chinese medicine.

* “1” for one drug of monotherapy; “2” for two drugs free combinations or single-pill combination consisted of two active ingredients; “>2” for >2 drugs free combinations or single-pill combination consisted of >2 active ingredients.

© 2022 Zhou H et al. *JAMA Network Open.*

eTable 6. Adverse Events in the Study

**Adverse Event [No. (%)]**	**Intervention (2,985)**	**Control (1,133)**
CVD Event		
AMI	NA	2(0.2)
CABG	4(0.1)	2(0.2)
Stroke	12(0.4)	3(0.3)
Death	13(0.4)	6(0.5)
Adverse Effect		
Cough	9(0.3)	NA
Stomach discomfort	3(0.1)	1(0.1)
Edema	3(0.1)	NA
Dizziness	1(0)	1(0.1)
Other	10(0.3)	2(0.2)

Abbreviations: AMI, acute myocardial infraction; CABG, coronary artery bypass graft; CVD, cardiovascular disease; NA, no data available.

© 2022 Zhou H et al. *JAMA Network Open.*

eTable 7. Sensitivity Analysis for Primary and Secondary Outcomes After Imputing the Missing Data in the Study								
	**Control**			**Intervention**			**Intervention effect(OR (95%CI))[a]**	***P value***
	**baseline**	**12 Month**	**Change from baseline**	**baseline**	**12 Month**	**Change from baseline**		
Mean SBP (SD), mm Hg	147.5(18.0)	145.5(18.0)	-2.0(-3.4 to-0.6) [b]	148.7(18.5)	137.0(13.5)	-11.8(-12.6 to -10.9) [b]	-9.7(-11.4 to -8.1)	<.001
Mean DBP (SD), mm Hg	85.5(11.3)	83.3(11.1)	-2.2(-3.1 to -1.4) [b]	85.0(11.5)	81.1(8.9)	-3.9(-4.4 to -3.4) [b]	-1.7(-2.7 to -0.7)	.001
BP control (%)	22.6	30.2	7.6(3.8-11.3) [b]	23.3	47.3	24.0(21.7-26.3) [b]	1.18(1.13-1.23)	<.001
BP classification (%)								
Stage 1 hypertension	42.7	42.1	-0.6(-4.5-3.3)	39.0	27.4	-11.6(-14.0 to -9.2) [b]	0.90(0.86-0.94)	<.001
Stage 2 hypertension	22.6	16.3	-6.4(-9.3 to -3.4) [b]	23.3	6.6	-16.7(-18.5 to -14.9) [b]	0.90(0.87-0.93)	<.001
Stage 3 hypertension	6.0	5.8	-0.2(-1.9-1.5)	7.1	1.1	-6.0(-7.0 to -4.9) [b]	0.94(0.93-0.96)	<.001
Lifestyle factors (%)								
Current smoking	14.2	14.9	0.7(-2.2-3.6)	15.7	14.6	-1.1(-2.9-0.7)	0.98(0.95-1.02)	.30
Current drinking	17.0	18.7	1.7(-1.4-4.8)	18.0	16.2	-1.8(-3.7-0.1)	0.96(0.93-1.00)	.05
Overweight/Obesity	73.4	70.1	-3.3(-7.1-0.5)	70.8	67.3	-3.5(-5.8 to -1.2) [b]	1.00(0.95-1.04)	.92
Regular exercise	42.5	56.3	13.8(9.8-17.9) [b]	45.2	59.8	14.7(12.2-17.1) [b]	1.01(0.96-1.06)	.73

Abbreviations: SBP, systolic blood pressure; DBP, diastolic blood pressure; OR, odds ratio. Data were presented as mean(SD) for continuous outcomes; proportion for dichotomous outcomes.

[a] Data were presented as mean(95%CI) for continuous outcomes, and OR(95%CI) for dichotomous outcomes.

[b] $P<0.05$.

© 2022 Zhou H et al. *JAMA Network Open.*

eFigure 1 CONSORT Flow Diagram

CONSORT
TRANSPARENT REPORTING of TRIALS

CONSORT 2010 Flow Diagram

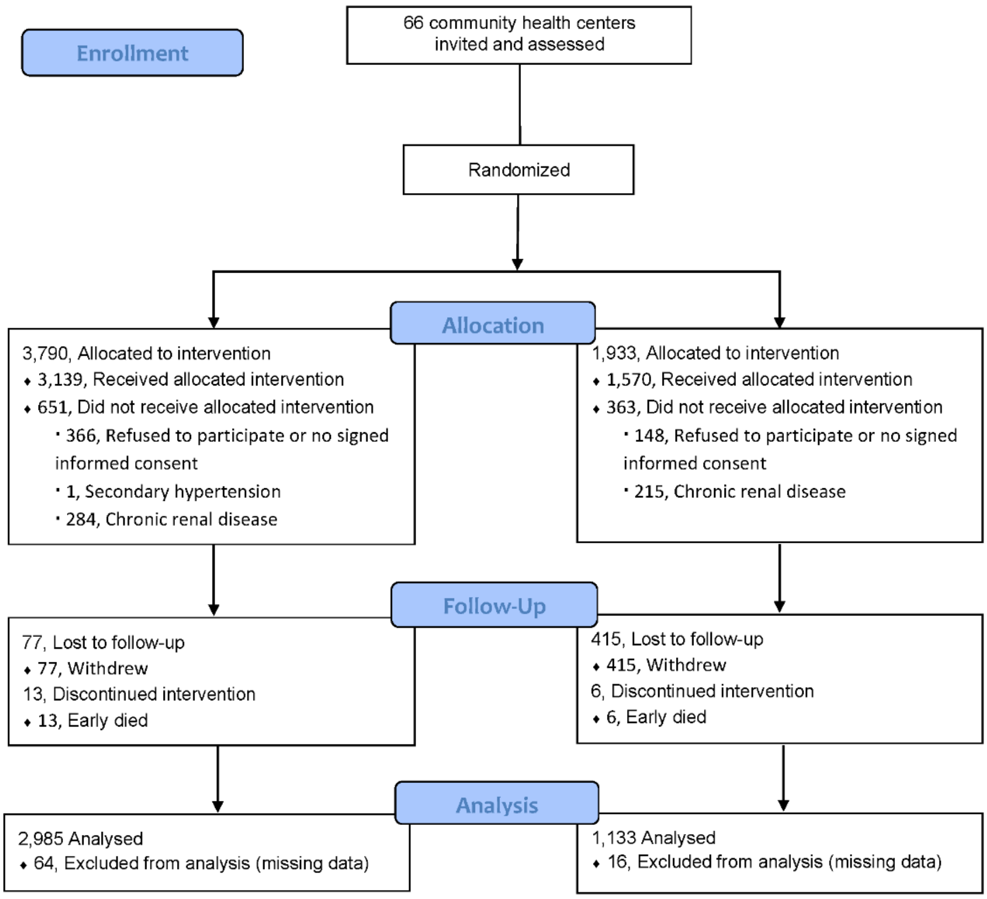

© 2022 Zhou H et al. *JAMA Network Open.*

eFigure 2 Overview of the Web-based platform

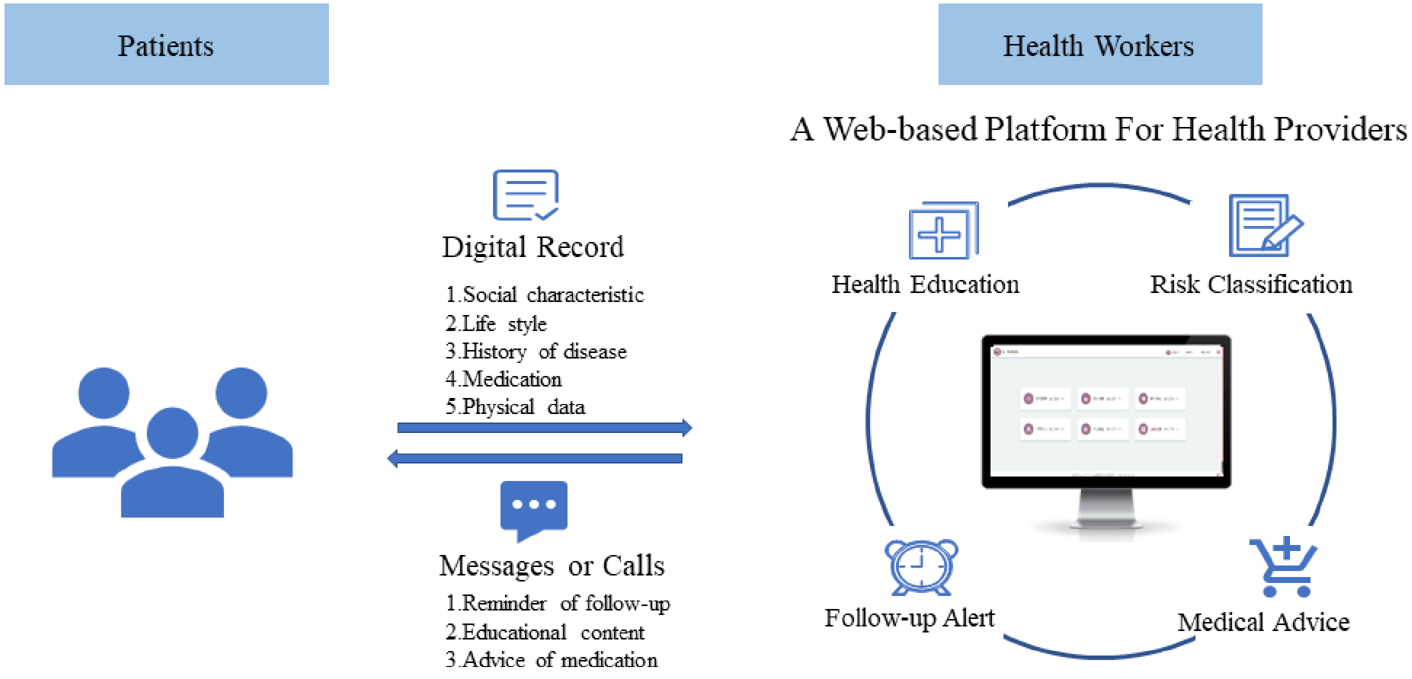

© 2022 Zhou H et al. *JAMA Network Open.*

eFigure 3 Subgroup Analysis for Change in Blood Pressure at 12 Months

A. Mean change in systolic blood pressure

Subgroup	Intervention No. of Patients	Intervention Mean Change (95%CI)	Control No. of Patients	Control Mean Change (95%CI)	Net Dfifference(95%CI)	Pvalue
**Sex**						
men	1330	−10.7(−11.9−−9.5)	523	−0.4(−2.3−1.5)	−10.3(−12.6−−8.1)	<.0001
women	1655	−12.7(−13.8−−11.5)	610	−3.3(−5.2−−1.4)	−9.4(−11.6−−7.1)	<.0001
**Age(y)**						
<65	1734	−11.9(−13.0−−10.8)	674	−0.5(−2.2−1.3)	−11.4(−13.5−−9.4)	<.0001
≥65	1251	−11.7(−13.0−−10.4)	459	−4.1(−6.3−−1.9)	−7.6(−10.1−−5.0)	<.0001
**Educational attainment**						
≤middle school	2292	−11.9(−12.9−−11.0)	861	−2.1(−3.7−−0.5)	−9.8(−11.7−−7.9)	<.0001
high school	629	−11.5(−13.2−−9.8)	249	−1.4(−4.1−1.2)	−10.1(−13.2−−6.9)	<.0001
≥colloge school	64	−9.5(−14.0−−4.9)	23	−2.7(−10.4−5.1)	−6.8(−15.8−2.2)	0.1368
**Region**						
west	838	−12.7(−14.3−−11.1)	324	−5.6(−8.2−−3.0)	−7.1(−10.1−−4.0)	<.0001
central	672	−11.1(−12.8−−9.5)	327	1.9(−0.5−4.2)	−13.0(−15.9−−10.2)	<.0001
east	1475	−11.7(−12.9−−10.4)	482	−2.1(−4.2−0.0)	−9.5(−12.0−−7.1)	<.0001
**BMI**						
normal	858	−11.5(−13.0−−10.0)	300	−2.0(−4.5−0.6)	−9.6(−12.6−−6.6)	<.0001
overweight	1280	−11.3(−12.5−−10.0)	509	−2.5(−4.5−−0.4)	−8.8(−11.2−−6.4)	<.0001
obesity	804	−12.7(−14.3−−11.0)	316	−0.5(−3.1−2.2)	−12.2(−15.3−−9.1)	<.0001
**CVD family history**						
no	1829	−11.7(−12.7−−10.6)	728	−2.0(−3.7−−0.3)	−9.7(−11.7−−7.7)	<.0001
yes	1156	−11.7(−13.3−−10.2)	405	−2.1(−4.5−0.4)	−9.7(−12.6−−6.8)	<.0001

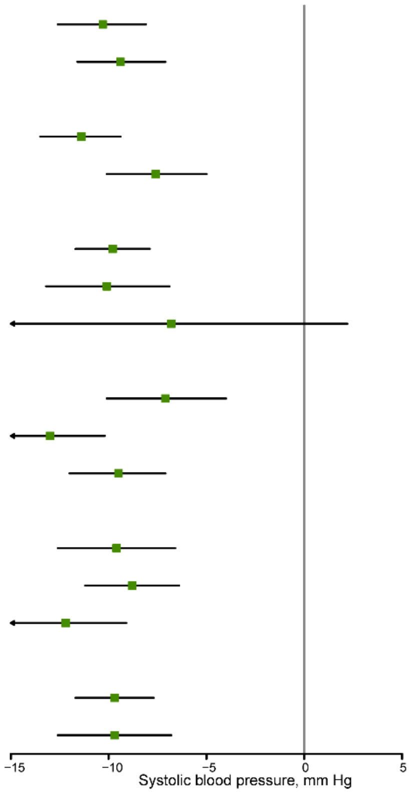

© 2022 Zhou H et al. *JAMA Network Open.*

B. Mean change in diastolic blood pressure

Subgroup	Intervention No. of Patients	Intervention Mean Change (95%CI)	Control No. of Patients	Control Mean Change (95%CI)	Net Dfifference(95%CI)	Pvalue
**Sex**						
men	1330	−5.1(−5.9−−4.3)	523	−1.5(−2.7−−0.2)	−3.6(−5.1−−2.1)	<.0001
women	1655	−3.0(−3.7−−2.3)	610	−2.9(−4.1−−1.7)	−0.1(−1.5−1.2)	0.8395
**Age(y)**						
<65	1734	−5.0(−5.7−−4.4)	674	−1.4(−2.5−−0.3)	−3.7(−4.9−−2.4)	<.0001
≥65	1251	−2.4(−3.2−−1.6)	459	−3.5(−4.8−−2.2)	1.1(−0.4−2.6)	0.148
**Educational attainment**						
≤middle school	2292	−3.5(−4.1−−2.9)	861	−2.9(−3.9−−1.9)	−0.7(−1.8−0.5)	0.2633
high school	629	−5.2(−6.4−−4.1)	249	−0.2(−2.0−1.7)	−5.1(−7.3−−2.9)	<.0001
≥colloge school	64	−6.3(−9.6−−3.1)	23	−1.1(−6.7−4.5)	−5.3(−11.8−1.2)	0.1112
**Region**						
west	838	−4.2(−5.2−−3.1)	324	0.1(−1.6−1.8)	−4.3(−6.2−−2.3)	<.0001
central	672	−4.3(−5.4−−3.3)	327	−3.6(−5.2−−2.1)	−0.7(−2.6−1.1)	0.4483
east	1475	−3.7(−4.4−−2.9)	482	−2.9(−4.2−−1.6)	−0.8(−2.3−0.7)	0.3119
**BMI**						
normal	858	−3.5(−4.4−−2.6)	300	−2.2(−3.7−−0.6)	−1.4(−3.2−0.5)	0.1461
overweight	1280	−3.7(−4.5−−2.9)	509	−2.2(−3.5−−1.0)	−1.4(−2.9−0.1)	0.0617
obesity	804	−4.6(−5.6−−3.5)	316	−1.8(−3.5−−0.1)	−2.8(−4.8−−0.8)	0.0069
**CVD family history**						
no	1829	−3.5(−4.2−−2.9)	728	−2.0(−3.0−−0.9)	−1.6(−2.8−−0.4)	0.0111
yes	1156	−4.0(−5.0−−3.1)	405	−2.8(−4.4−−1.2)	−1.2(−3.1−0.6)	0.192

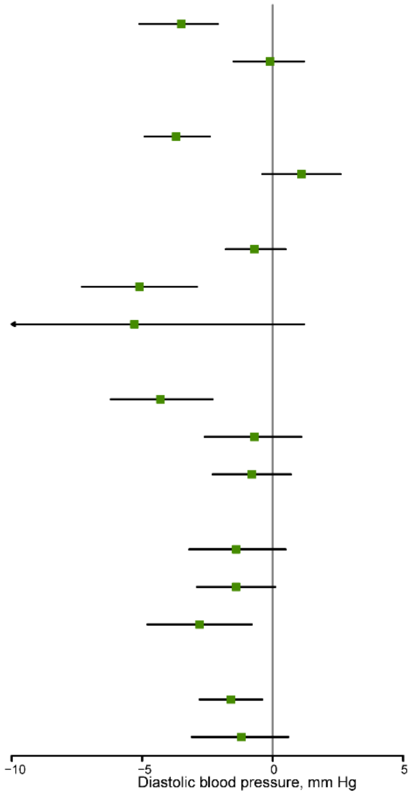

© 2022 Zhou H et al. *JAMA Network Open.*

eFigure 4 Mean Change in Lifestyle Factors over Time

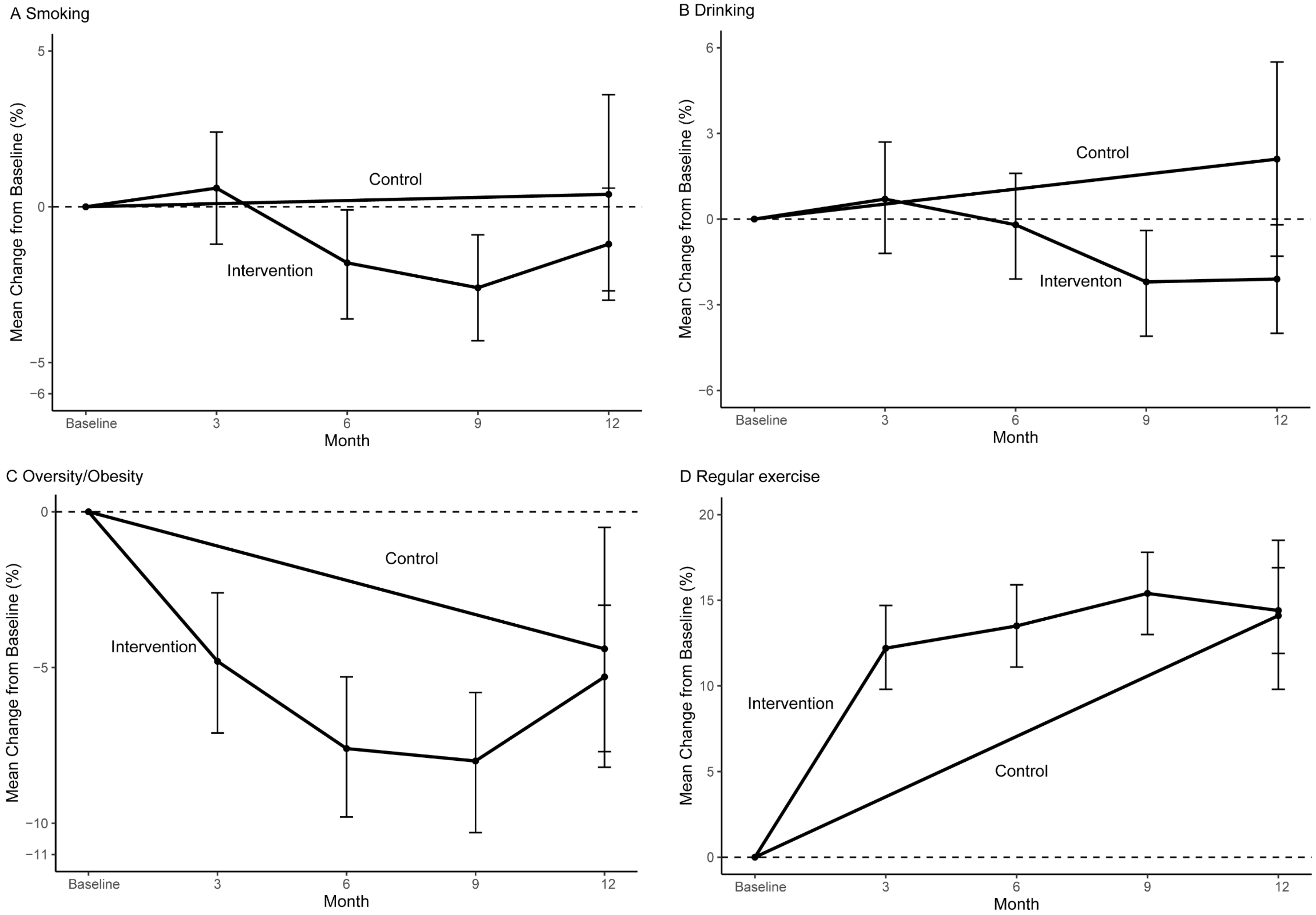

© 2022 Zhou H et al. *JAMA Network Open.*
