# Supplementary material for: Effect of a Multicomponent Intervention Delivered on a Web-Based Platform on Hypertension Control: A Cluster Randomized Clinical Trial
Source: JAMA Netw Open. 2022 Dec 7;5(12):e2245439. doi: 10.1001/jamanetworkopen.2022.45439 (PMC9856259; doi:10.1001/jamanetworkopen.2022.45439)
Supplement: Supplement 3. — Data Sharing Statement [file jamanetwopen-e2245439-s003.pdf]

Data Sharing Statement

Zhou. Effect of a Multicomponent Intervention Delivered on a Web-Based Platform on Hypertension Control. *JAMA Netw Open*. Published December 07, 2022. doi:10.1001/jamanetworkopen.2022.45439

Data
Data available: Yes
Data types: Deidentified participant data
How to access data: wangznegwu@foxmail.com
When available: With publication

Supporting Documents
Document types: Informed consent form
How to access documents: wangznegwu@foxmail.com
When available: With publication

Additional Information
Who can access the data: Zengwu Wang
Types of analyses: For results verification
Mechanisms of data availability: with investigator support AND with asigned data access agreement
